# Measurement of ^15^N longitudinal relaxation rates in ^15^NH_4_^+^ spin systems to characterise rotational correlation times and chemical exchange

**DOI:** 10.1016/j.jmr.2017.01.015

**Published:** 2017-06

**Authors:** D. Flemming Hansen

**Affiliations:** Institute of Structural and Molecular Biology, Division of Biosciences, University College London, London WC1E 6BT, United Kingdom

**Keywords:** AX_4_ spin systems, NMR, Nuclear spin relaxation, Ammonium, Chemical exchange

## Abstract

Many chemical and biological processes rely on the movement of monovalent cations and an understanding of such processes can therefore only be achieved by characterising the dynamics of the involved ions. It has recently been shown that ^15^N-ammonium can be used as a proxy for potassium to probe potassium binding in bio-molecules such as DNA quadruplexes and enzymes. Moreover, equations have been derived to describe the time-evolution of ^15^N-based spin density operator elements of ^15^NH_4_^+^ spin systems. Herein NMR pulse sequences are derived to select specific spin density matrix elements of the ^15^NH_4_^+^ spin system and to measure their longitudinal relaxation in order to characterise the rotational correlation time of the ^15^NH_4_^+^ ion as well as report on chemical exchange events of the ^15^NH_4_^+^ ion. Applications to ^15^NH_4_^+^ in acidic aqueous solutions are used to cross-validate the developed pulse sequence while measurements of spin-relaxation rates of ^15^NH_4_^+^ bound to a 41 kDa domain of the bacterial Hsp70 homologue DnaK are presented to show the general applicability of the derived pulse sequence. The rotational correlation time obtained for ^15^N-ammonium bound to DnaK is similar to the correlation time that describes the rotation about the threefold axis of a methyl group. The methodology presented here provides, together with the previous theoretical framework, an important step towards characterising the motional properties of cations in macromolecular systems.

## Highlights

• A pulse sequence is derived to select ^15^N coherences within the ^15^NH_4_^+^ spin-system.
• A sequence for measurement of ^15^N spin-relaxation rates in ^15^NH_4_^+^ is presented.
• The effective correlation time of ^15^NH_4_^+^ bound to a 41 kDa protein is characterised.

## Graphical abstract

**Figure:**
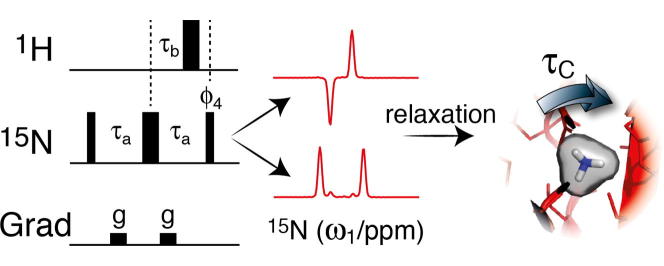

## 1 Introduction

Monovalent cations such as potassium and sodium regulate many enzymes via binding to either active sites or to allosteric sites [1], [2], [3]. The dynamics and movements of these ions are therefore crucial factors to understand the regulation of enzymes by monovalent cations and experimental insight into the dynamics of cations therefore becomes important in order to characterise many biological processes. Solution NMR spectroscopy is a powerful technique to probe the dynamics of nuclear spin, where in particular nuclear spin-relaxation rates have been used to report on the dynamics of small ions [4], [5], [6], [7] to large macromolecular complexes [8], [9], [10], [11]. The obtained nuclear spin-relaxation rates often report on both the rotational correlation time of the nuclear spin as well as on chemical exchange between different magnetic environments and a separation of the different contributions to the observed nuclear spin-relaxation rate is thus important. Several strategies have therefore previously been employed to separate the different contributions to spin-relaxation rates, including, measurements at several magnetic field strengths [12], measurements of cross-correlated relaxation rates [13], [14], and measurements of the relaxation rate of related anti-phase coherences [15], [16].

Recently it was shown that ^15^NH_4_^+^ can be used as a proxy for potassium to probe potassium-binding sites in nucleic acids and enzymes [17], [18], [19], [20]. This method relies on several characteristics of the ammonium ion: (i) The ionic radius of the ammonium ion is similar to the ionic radius of potassium, 1.44 Å *versus* 1.33 Å [21], [22], such that ammonium generally binds to potassium binding-sites in macromolecules. (ii) Under physiological conditions the chemical exchange of the ammonium protons with the bulk solvent is so fast that free ammonium is not observed in NMR correlation spectra, however, the protection of the ammonium ion, for example by a protein environment, slows the exchange of the protons with the bulk solvent to such an extend that these are observed in two-dimensional ^15^N-^1^H correlation spectra [19]. (iii) Finally, protein-bound ammonium ions appear to have a fast internal correlation time such that the line broadening due to the ^15^N-^1^H dipolar-dipole interactions is limited.

The recently developed theory for ^15^N spin relaxation in ^15^NH_4_^+^ spin systems together with the possibility of obtaining ^15^N-^1^H correlation maps of protein-bound ^15^NH_4_^+^ open up the possibility of quantifying the dynamics of ammonium ions, even within potassium binding-sites of large proteins, and thus for correlating cation dynamics with macromolecular function. Given the current development of techniques to probe ammonium ions in proteins and nucleic acids it is therefore of interest to derive methods to experimentally measure nuclear spin-relaxation rates to report on the rotational correlation time and chemical exchange of ammonium ions. The advantage of the ^15^NH_4_^+^ spin system is the availability of a wealth of coherences and spin density elements, whose relaxation rates each report differently on the rotational correlation time and chemical exchange. Herein NMR pulse sequences are developed firstly to select different spin density matrix elements and secondly to measure ^15^N-based longitudinal relaxation rates of the ^15^NH_4_^+^ spin system. Applications to ^15^N-ammonium in an acidic aqueous solution and ^15^N-ammonium bound in a potassium binding-site of a ∼41 kDa domain of the protein DnaK are presented to illustrate the general utility of the derived pulse sequences.

## 2 Theory

*Time-evolution of the ^15^NH_4_^+^ spin systems:* The time evolution of a spin system is generally given by the Liouville-von-Neumann equation [23], [24], [25]:

(1) dσ(t)dt=-i[H^0,σ(t)]-Γ(σ(t)-σeq)

where H^0 is the time independent part of the spin-Hamiltonian, σ_eq_ is the equilibrium density operator, and Γ is the spin-relaxation super-operator, which was derived previously for the ^15^N-ammonium spin-system [20]. The time-independent part of the Hamiltonian is here given by:

(2) H^0=H^Z+H^J

where the total Zeeman Hamiltonian is, H^Z=(Hz1+Hz2+Hz3+Hz4)ωH+NzωN, and the ^15^N-^1^H scalar-coupling Hamiltonian is given by H^J=πJ(2Hz1Nz+2Hz2Nz+2Hz3Nz+2Hz4Nz), and *J* is the nitrogen-proton one-bond scalar coupling constant.

As shown previously, there are nine single quantum ^15^N transitions within the tetrahedral ^15^NH_4_^+^ spin system, however, due to the tetrahedral symmetry and degeneracy of the ^15^NH_4_^+^ spin system there are only five characteristic frequencies of the single quantum ^15^N coherences: −4π*J* + Ω, −2π*J* + Ω, Ω, 2π*J* + Ω, 4π*J* + Ω, where Ω is the offset from the RF carrier. The frequency of a ^15^N transition depends on the spin states of the four ammonium protons, for example, when all the protons are in the α state, the frequency is 4π*J* + Ω. In turn, the spin state of the four protons can be described using different basis sets; for example a Zeeman basis given by the eigenfunctions to the Zeeman Hamiltonian or a Cartesian basis. In the Zeeman basis set the transitions fall in three spin manifolds, *A*_1_, *T*_2_, and *E* according to the *T*_d_ symmetry of the ammonium ion as discussed previously [20]. For the development of the pulse sequence below the spin densities of the four ammonium protons are most conveniently described using the product operator formalism [26] and the Cartesian basis set.

In the product operator formalism the equilibrium density operator of the ammonium ion is given by σ_eq_ ∝ γ_H_(*H*_z1_ + *H*_z2_ + *H*_z3_ + *H*_z4_) + γ_N_*N*_z_, where γ_H_ and γ_N_ are the gyromagnetic ratio of the proton and the nitrogen nuclear spin, respectively, and *H*_z_*_i_* (*i* = 1, 2, 3, 4) and *N*_z_ are the Cartesian product operator describing the longitudinal magnetisation of the four protons and the nitrogen spin, respectively. The transverse nitrogen magnetisation is written as *N*_+_ = *N*_x_ + *i N*_y_, where *i* is the imaginary unit. Thus, using the product operator formalism the nine single quantum nitrogen transitions are described by $B_{\text{xyz}}$ ={*N*_+_, 2*N*_+_***H***_z_, 4*N*_+_***H***_z_***H***_z_, 8*N*_+_***H***_z_***H***_z_***H***_z_, 16*N*_+_***H***_z_***H***_z_***H***_z_***H***_z_, *N*_+_***H***_+_***H***_−_, 2*N*_+_***H***_+_***H***_−_***H***_z_, 4*N*_+_***H***_+_***H***_−_***H***_z_***H***_z_, *N*_+_***H***_+_***H***_−_***H***_+_***H***_−_} in the Cartesian basis set, where the following notation has been used: ***H***_z_ = *H*_z1_ *+* *H*_z2_ *+* *H*_z3_ *+* *H*_z4_; ***H***_z_***H***_z_ = *H*_z1_*H*_z2_ + *H*_z1_*H*_z3_ + *H*_z1_*H*_z4_ + *H*_z2_*H*_z3_ + *H*_z2_*H*_z4_ + *H*_z3_*H*_z4_; ***H***_z_***H***_z_***H***_z_ = *H*_z1_*H*_z2_*H*_z3_ + *H*_z1_*H*_z2_*H*_z4_ + *H*_z1_*H*_z3_*H*_z4_ + *H*_z2_*H*_z3_*H*_z4_; ***H***_z_***H***_z_***H***_z_***H***_z_ = *H*_z1_*H*_z2_*H*_z3_*H*_z4_; ***H***_+_***H***_−_ *=* $\sum_{i\neq j}H_{+\text{,}i}H_{-\text{,}j}$; ***H***_+_***H***_−_***H***_z_ *=* $\sum_{i\neq j\neq k}H_{+\text{,}i}H_{-\text{,}j}H_{z\text{,}k}$; ***H***_+_***H***_−_***H***_z_***H***_z_ *=* $\sum_{i\neq j\neq k\neq l}H_{+\text{,}i}H_{-\text{,}j}H_{z\text{,}k}H_{z\text{,}l}$; ***H***_+_***H***_−_***H***_+_***H***_−_ *=* $\sum_{i\neq j\neq k\neq l}H_{+\text{,}i}H_{-\text{,}j}H_{+\text{,}k}H_{-\text{,}l}$, The advantage of using the Cartesian basis set here is that spin density elements present during the pulse sequence are more easily identified and the relaxationoftheproton spins by external sources is conveniently implemented as additions to the auto-relaxation rates as described previously [20].

The evolution of spin density matrix elements under the scalar coupling Hamiltonian forms the basis for a separation of different nitrogen single quantum coherences. It is therefore of interest to characterise how the spin density elements of the basis B_xyz_ evolve under the scalar coupling Hamiltonian. Only considering scalar coupling and ignoring relaxation, it was shown previously that [20]

**Figure:**
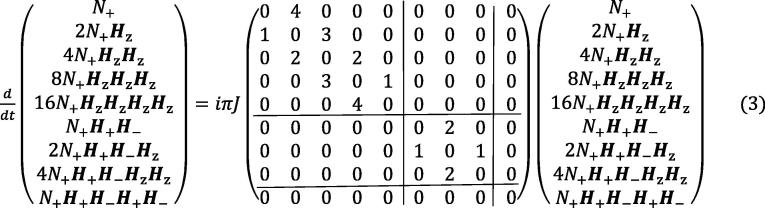

Firstly it is noted that, due to the symmetry of the ammonium ion, the scalar coupling Hamiltonian is only mixing coherences *within* three groups: {*N*_+_, 2*N*_+_***H***_z_, 4*N*_+_***H***_z_***H***_z_, 8*N*_+_***H***_z_***H***_z_***H***_z_, 16*N*_+_***H***_z_***H***_z_***H***_z_***H***_z_,}, {*N*_+_***H***_+_***H***_−_, 2*N*_+_***H***_+_***H***_−_***H***_z_, 4*N*_+_***H***_+_***H***_−_***H***_z_***H***_z_}, and *N*_+_***H***_+_***H***_−_***H***_+_***H***_−_ (*N*_+_***H***_+_***H***_−_***H***_+_***H***_−_ commutes with the scalar coupling Hamiltonian). Thus, starting from in-phase transverse nitrogen magnetisation, *N*_x_, gives:

(4) $$N_{x}\overset{\tau 2\pi JH_{z}N_{z}}{\to }\frac{1}{8}(3+4\cos2\pi J\tau +\cos4\pi J\tau )N_{x}+\frac{1}{8}(2\sin2\pi J\tau +\sin4\pi J\tau )2N_{y}H_{z}-\frac{1}{8}(1-\cos4\pi J\tau )4N_{x}H_{z}H_{z}-\frac{1}{8}(2\sin2\pi J\tau -\sin4\pi J\tau )8N_{y}H_{z}H_{z}H_{z}+\frac{1}{8}(3-4\cos2\pi J\tau +\cos4\pi J\tau )16N_{x}H_{z}H_{z}H_{z}H_{z}$$

Eq. (4) implies that evolution of *N*_x_ for a period of 1/(2*J*) generates the quadruple anti-phase coherence 16*N*_x_***H***_z_***H***_z_***H***_z_***H***_z_. Moreover, because the single anti-phase coherence, 2*N_x,y_****H***_z_, is easily generated from the equilibrium density operator it’s time evolution is also of particular interest here:

(5) $$2N_{x}H_{z}\overset{\tau 2\pi JH_{z}N_{z}}{\to }\frac{1}{2}(2\sin2\pi J\tau +\sin4\pi J\tau )N_{y}+\frac{1}{2}(\cos2\pi J\tau +\cos4\pi J\tau )2N_{x}H_{z}+\frac{1}{2}(\sin4\pi J\tau )4N_{y}H_{z}H_{z}-\frac{1}{2}(\cos2\pi J\tau -\cos4\pi J\tau )8N_{x}H_{z}H_{z}H_{z}-\frac{1}{2}(2\sin2\pi J\tau -\sin4\pi J\tau )16N_{y}H_{z}H_{z}H_{z}H_{z}$$

Eqs. (4), (5) imply that by choosing appropriate values of τ in a pulse sequence a series of different coherences can be generated by evolving either in-phase transverse *N*_x_ magnetisation or the single anti-phase coherence 2*N*_x_***H***_z_.

It should be noted that inclusion of proton spin-flip, *R*_1_(***H***_z_), in the evolution described in Eq. (3) causes differential relaxation of the ^15^N single quantum Cartesian density elements and can effectively be viewed as a five-site exchange between the transitions of the ^15^NH_4_^+^ multipliet. Thus, the signal with a frequency of −4π*J* + Ω exchanges magnetisation with the signal at −2π*J* + Ω, the signal with a frequency of −2π*J* + Ω exchanges with the signals at −4π*J* + Ω and at Ω, and so forth. The consequence of proton spin-flip can therefore be a change in the characteristic frequencies, linewidths and intensities, which in turn can be calculated from the eigenvalues and eigenvectors of a Liouvillian that includes both the scalar coupling and proton spin-flip, *R*_1_(***H***_z_). It can be shown that inclusion of *R*_1_(***H***_z_) in the Liouvillian, and assuming that *R*_1_(***H***_z_) < 2π*|J|*, changes the characteristic frequencies to $\left\{-4\pi J{\left[1-\zeta^{2}\right]}^{1/2}+\Omega \text{,}-2\pi J{\left[1-\zeta^{2}\right]}^{1/2}+\Omega \text{,}\Omega \text{,}2\pi J{\left[1-\zeta^{2}\right]}^{1/2}+\Omega \text{,}4\pi J{\left[1-\zeta^{2}\right]}^{1/2}+\Omega \right\}$, where $\zeta =R_{1}(H_{z})/(2\pi J)\text{.}$ Moreover, when the single anti-phase coherence 2*N*_+_***H***_z_ is excited and detected, for example in a standard coupled ^15^N-^1^H correlation spectrum, the intensity ratio of the five signals is 1:*r*:0:*r*:1, with $r=1-5\zeta \left(\zeta -\frac{4i}{5}\sqrt{1-\zeta^{2}}\right)$. For experimental spectra of ^15^NH_4_^+^ the effects on the frequencies and the intensity ratios are generally small. For example, when *J* = −70 Hz and *R*_1_(***H***_z_) = 35 s^−1^, which is well within the range of the data shown below, the characteristic frequencies are {−139.56 Hz, −69.79 Hz, 0 Hz, 69.79 Hz, 139.56 Hz} and the absolute intensity ratio is 1:1.02:0:1.02:1. It should be noted that since *r* is a complex number, the proton spin-flip also causes a relative phase-shift of the signals within the multiplet, as also observed previously for two-site chemical exchange [27]; this phase-shift is approximately 10° for the example above.

## 3 Results

**Fig. 1:**
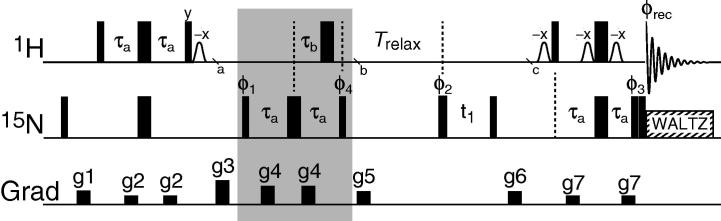
Pulse sequence to measure longitudinal relaxation rates of individual spin density matrix elements of the ^15^NH_4_^+^ spin-system. The ^1^H carrier is placed on the water and the ^15^N carrier is placed in the middle of the ^15^NH_4_^+^ region (∼20 ppm). All pulses are applied at the highest possible power levels, with the exception of the ^1^H water selective pulses (open bell-shaped pulses) and the ^15^N decoupling, where a ∼350 Hz field strength and a 1.5 kHz WALTZ-16 decoupling [28] are employed, respectively. The element immediately following the initial INEPT (grey box) is used to select different coherences of the ^15^NH_4_^+^ spin system, as detailed in the text. The delay used are: τ_a_ = 3.47 ms and τ_b_ varied as described in the main text. Pulses without annotation are applied with *x*-phase. The phase cycle is: φ_1_ = *x*, −*x*, φ_2_ = 2(*x*), 2(−*x*), φ_3_ = 4(*x*), 4(−*x*), φ_rec_ = *x*, −*x*, −*x*, *x*. The phase of φ_4_ is chosen as a part of the coherence selection element and detailed in the text and in Fig 3. Quadrature detection in the indirect ^15^N dimension is achieved by altering ϕ_2_ and ϕ_rec_ in the States-TPPI manner. Gradients are used to remove artifacts and are applied using a sine bell-shaped profile for 0.5 ms with maximum strengths of, g1: 24 G/cm, g2: 7.3 G/cm, g3: 35 G/cm, g4: 15 G/cm, g5: 8.6 G/cm, g6: 11.2 G/cm, g7: 8.6 G/cm.

*Pulse sequence for measuring the longitudinal relaxation rates of ^15^NH_4_^+^:* ^15^N-ammonium ions in an acid aqueous solution or bound to proteins or nucleic acids can be characterised by ^15^N-^1^H correlations spectra [17], [18], [19], [20]. Such two-dimensional spectra can be obtained using standard ^15^N-^1^H correlation experiments, where after an initial INEPT, 90*_x_*(^1^H) – τ – 180*_x_*(^1^H), 180*_x_*(^15^N) – τ – 90_y_(^1^H) with τ = 1/(4*J*), a spin density operator proportional to 2*N*_z_***H***_z_ is generated. Subsequently a 90*_x_*(^15^N) pulse generates −2*N*_y_***H***_z_ for chemical shift evolution and a final INEPT transfers anti-phase 2*N*_x,y_***H***_z_ magnetisation to transverse proton magnetisation for detection. When the ^15^N-^1^H scalar coupling is allowed to evolve during the 2*N_x,y_****H***_z_ chemical shift evolution period signals at the five ^15^N characteristic frequencies −4π*J* + Ω, −2π*J* + Ω, Ω, 2π*J* + Ω, and 4π*J* + Ω (Eq. (5)) are observed with an intensity ratio of 1:1:0:1:1 [20] in the limit where *R*_1_(^1^H) ≲ 2π*|J|*(see Section 2). The pulse sequence used here for generating subspectra of the ^15^NH_4_^+^ quintet and for measuring relaxation rates of the longitudinal density operator elements is shown in Fig. 1 and is based on the ^15^N-^1^H correlation experiment described above.

The sequence in Fig. 1 differs from a standard ^15^N-^1^H correlation experiment by the insertion of a selection element between *a* and *b* and a relaxation delay prior to the ^15^N chemical shift evolution. The final INEPT between *c* and acquisition selects for the two spin-order longitudinal spin density element, 2*N*_z_***H***_z_, and transfers it to ***H***_y_ magnetisation for detection while a pair of ^15^N 90° pulses, with the first pulse phase-cycled *x*, −*x*, is placed immediately prior to acquisition to remove anti-phase 2*N*_z_***H***_xy_ coherences.

**Fig. 2:**
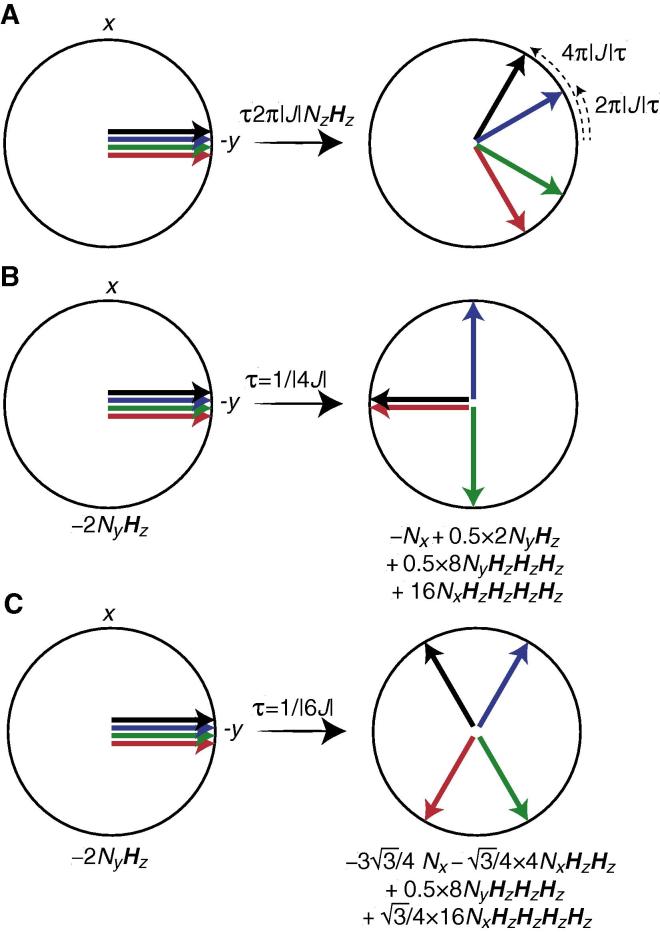
(A) Vector diagram showing the evolution of transverse ^15^N magnetisations of the ^15^NH_4_^+^ spin-system under the ^15^N-^1^H scalar coupling Hamiltonian. The vectors correspond to the four transitions observed in the coupled ^15^N spectrum that is obtained by exciting and detecting the anti-phase coherence 2*N*_xy_***H***_z_. Specifically, the red vector corresponds to the transition where all the ammonium protons are in the α-state, green vector corresponds to the transitions where three of the protons are in the α-state, blue vector corresponds to the transitions where one of the protons is in the α-state, and black vector corresponds to the situation where all the protons are in the β-state. (B) Evolution of the anti-phase coherence −2*N*_y_***H***_z_ under the scalar coupling Hamiltonian for a time of |1/4*J*| (*J* < 0 for ^15^N-^1^H scalar couplings). Selection along the *y*-axis generates an intensity ratio of 0:−1:0:1:0 of the five ^15^N characteristic frequencies and a spin density element proportional to *N*_z_−16*N*_z_***H***_z_***H***_z_***H***_z_***H***_z_, while selection along the *x*-axis generates an intensity ratio of 1:0:0:0:1 of the five ^15^N characteristic frequencies and a spin density element proportional to 2*N*_z_***H***_z_ + 8*N*_z_***H***_z_***H***_z_***H***_z_. (C) Evolution of the anti-phase coherence −2*N*_y_***H***_z_ under the scalar coupling Hamiltonian for a time of |1/6*J*|. Selection along the *y*-axis generates an intensity ratio of 1:1:0:−1:−1 of the five ^15^N characteristic frequencies, while selection along the *x*-axis generates an intensity ratio of 1:−1:0:−1:1 of the five ^15^N characteristic frequencies.

*Selecting subspectra of the ^15^NH_4_^+^ quintet:* The initial 90_ϕ1_^15^N pulse of the selection element between *a* and *b* in Fig. 1 generates the ±2*N*_y_***H***_z_ anti-phase spin density element. Free precession of 2*N*_y_***H***_z_ during the coherence selection element can be characterised schematically using a vector model, where each of the four observed signals of the ^15^NH_4_^+^ quintet are represented by a vector (Fig. 2). A ^15^N 180° pulse is applied in the middle of the coherence selection element such that chemical shift evolution of ^15^N is refocused. Relaxation during the selection element is disregarded initially and therefore only the evolution under the ^15^N-^1^H scalar coupling Hamiltonian is considered immediately below.

The selection element shown in the pulse sequence of Fig. 1 relies on both a variation of the delay τ_b_ and on the phase ϕ_4_. In the limiting case when τ_b_ = τ_a_ the evolution under the scalar coupling Hamiltonian is refocused, while when τ_b_ = 0 the scalar coupling evolves for 1/|2*J*| and a spin density element proportional to 8*N*_z_***H***_z_***H***_z_***H***_z_ is obtained (Eq. (5)). For other values of τ_b_ the evolution of the scalar coupling can conveniently be followed using a vector diagram as shown in Fig.2A. Specifically, Fig.2B and C show the selection of four different spin density elements of the ^15^NH_4_^+^ spin-system. It should be noted that differential relaxation of the density elements {*N*_+_, 2*N*_+_***H***_z_, …, 16*N*_+_***H***_z_***H***_z_***H***_z_***H***_z_} during the selection element becomes significant for application to ^15^NH_4_^+^ systems with large proton spin-flip rates, *R*_1_(***H***_z_). As discussed below the effect of differential relaxations can be taken into account in the analysis of relaxation decay curves by introducing model parameters that describe the state immediately following the selection element.

**Fig. 3:**
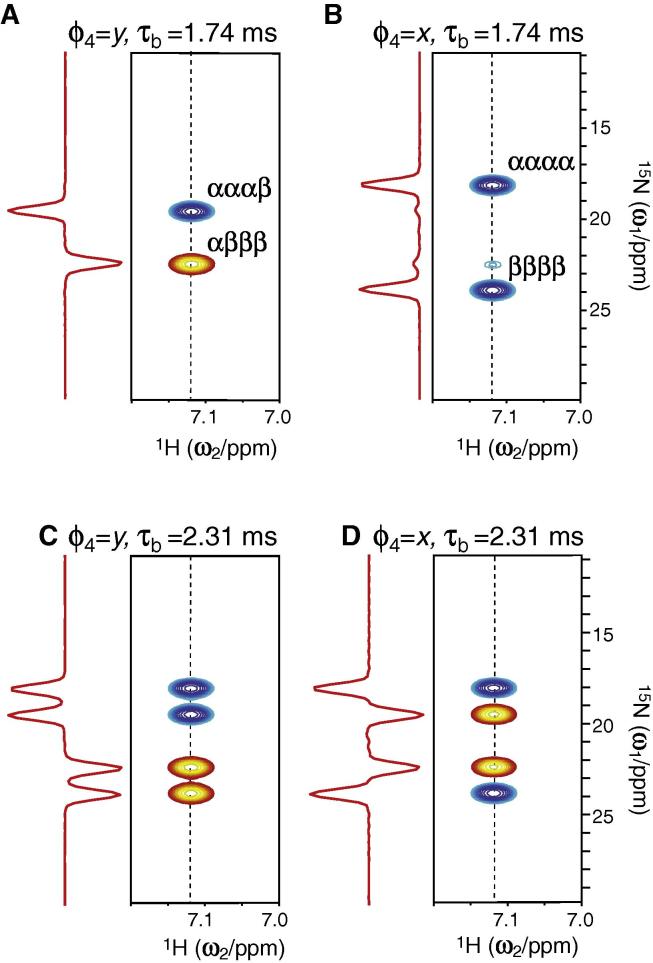
Selection of spin density matrix elements of ^15^NH_4_^+^ dissolved in an acidic aqueous solution (pH = 2.82). The spectra were obtained using the sequence shown in Fig 1, with *T*_relax_ = 0 and τ_b_ and ϕ_4_ as shown above each of the panels. (A) The selection of *N*_x_ − 16*N*_x_***H***_z_***H***_z_***H***_z_***H***_z_ coherences; corresponding to Fig2B with selection along *x*. (B) The selection of 2*N*_x_***H***_z_ + 8*N*_x_***H***_z_***H***_z_***H***_z_ coherences; corresponding to Fig2B with selection along the *y*-axis. (C) and (D) Selection shown in Fig2C with selection phase along the *x*- and *y*-axis, respectively.

The pulse sequence in Fig. 1 and the selection element were initially verified using ^15^NH_4_^+^ in an acidic aqueous solution. Under acidic conditions (pH = 2.82) the exchange rate of the ammonium protons with the water is slowed to an extent where ^15^N-^1^H correlation spectra can be obtained and where the pulse sequence in Fig. 1 can be verified. Fig. 3 shows the generation of four different spectra obtained by varying τ_b_ and ϕ_4_. The obtained spectra are in excellent agreement with the predicted ratio of the intensities of the multiplet structure.

Linear combinations of the spectra in Fig. 3 can be used to generate spectra of four of the individual lines of the ^15^NH_4_^+^ quintet, where for example, the spectra in Fig.3A–C can be used to generate a spectrum only consisting of the αααα line. It should be noted however that although spectra of the four lines can be generated, pure spectra of all of the five Cartesian ^15^N single quantum spin density elements, *N*_+_, 2*N*_+_***H***_z_, …, 16*N*_+_***H***_z_***H***_z_***H***_z_***H***_z_, cannot be generated using the described sequence. A spectrum of pure 2*N*_+_***H***_z_ and pure 8*N*_+_***H***_z_***H***_z_***H***_z_ can be generated from Fig.3B and D, or from spectra with τ_b_ = τ_a_ and τ_b_ = 0 s, respectively. A further consequence is that the intensity of the four lines, αααα, αααβ, αβββ, and ββββ can be used to derive the relative intensity of 2*N*_+_***H***_z_ and 8*N*_+_***H***_z_***H***_z_***H***_z_. On the contrary, the pure spectra corresponding to the three spin density elements *N*_+_, 4*N*_+_***H***_z_***H***_z_, and 16*N*_+_***H***_z_***H***_z_***H***_z_***H***_z_ are correlated such that their individual intensities cannot be separated using the selection elements in Fig. 1. Such a correlation is the result of an underdetermined system where four lines are observed in the coupled ^15^N-^1^H NMR spectra, however originating from five spin density elements. On the other hand, when the contributions from the five possible spin density elements are know, the intensities of the four lines can be calculated.

*Measuring longitudinal relaxation rates:* In general, the relaxation rate of the five longitudinal spin density elements, *N*_z_, 2*N*_z_***H***_z_, 4*N*_z_***H***_z_***H***_z_, 8*N*_z_***H***_z_***H***_z_***H***_z_ and 16*N*_z_***H***_z_***H***_z_***H***_z_***H***_z_ are different, since they have different contributions from the spectral density function *J*(ω) and different contributions from the proton spin-flip rate, *R*_1_(***H***_z_) [20]. When the rotational diffusion of the ^15^NH_4_^+^ ion is described accurately by one correlation time, τ_C_, the five relaxation rates, *R*_1_(*N*_z_), *R*_1_(2*N*_z_***H***_z_), *R*_1_(4*N*_z_***H***_z_***H***_z_), *R*_1_(8*N*_z_***H***_z_***H***_z_***H***_z_), and *R*_1_(16*N*_z_***H***_z_***H***_z_***H***_z_***H***_z_), as well as cross-correlated relaxations between the longitudinal spin density elements, only depend on two parameters, that is, the rotational correlation time τ_C_ and the proton spin-flip rate, *R*_1_(***H***_z_). Thus, as described below using the Liouvillian in Table 4 of Werbeck and Hansen [20], which includes both auto- and cross-correlated relaxations, the *two parameters* τ_C,eff_ and *R*_1_(***H***_z_) can be obtained from the intensities of the *four lines* observed in coupled ^15^NH_4_^+^ spectra recorded for different values *T*_relax_.

A relaxation delay, *T*_relax_, is inserted after the selection element and before the ^15^N chemical shift evolution in the sequence in Fig. 1, which allows the relaxation decay of the four lines {αααα, αααβ, αβββ, ββββ} to be obtained in order to characterise the dynamics of the ammonium ion. Initially ^15^NH_4_^+^ in acidic aqueous solutions (pH 2.82) was used as a model system. For such a system the proton spin-flip rate *R*_1_(***H***_z_) is given by the off-rate of the ammonium protons with the bulk solvent: ${}^{15}{\text{NH}}_{4}^{+}+{\text{H}}_{2}^{*}\text{O}\overset{k_{\text{ex}}}{\to }{}^{15}{\text{NH}}^{*}{\text{H}}_{3}^{+}+{\text{H}}^{*}\text{OH}$.

**Fig. 4:**
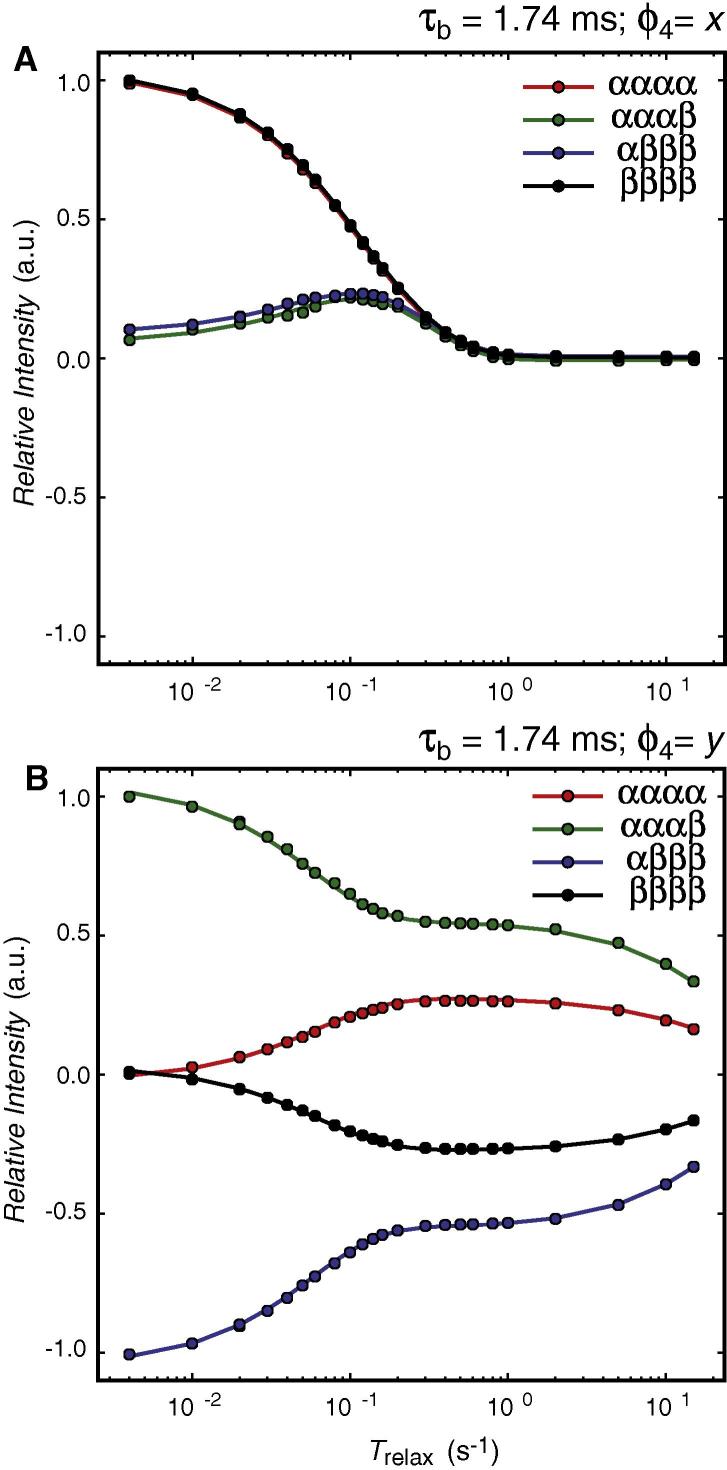
Decay curves of the four lines observed in the coupled ^15^NH_4_^+^ spectrum of ^15^NH_4_^+^ in an acidic aqueous solution; pH 2.82 at 278 K, and recorded at a static magnetic field of 11.74 T. The solid lines are obtained from best-fits of all the data shown to an evolution of the Liouvillian describing the decay of the five Cartesian longitudinal spin density elements (see text). (A) Decay curves obtained after selecting for 2*N*_z_***H***_z_ + 8*N*_z_***H***_z_***H***_z_***H***_z_ and using the pulse sequence of Fig 1. (B) Decay curves obtained after selecting for *N*_z_ −16*N*_z_***H***_z_***H***_z_***H***_z_***H***_z_. The double sigmoidal shape of the decay curves in the logarithmic plot shows first the faster relaxation of the quadruple anti-phase spin density element 16*N*_z_***H***_z_***H***_z_***H***_z_***H***_z_ followed by a substantially slower relaxation of the in-phase longitudinal magnetisation *N*_z_.

Although there are many possible combinations of τ_b_ and ϕ_4_, each allowing for a different selection of spin density elements, focus below is on selecting two initial states, that is (i) 2*N*_z_***H***_z_ + 8*N*_z_***H***_z_***H***_z_***H***_z_ and (ii) *N*_z_ −16*N*_z_***H***_z_***H***_z_***H***_z_***H***_z_. The decay curves obtained for ^15^NH_4_^+^ in an acidic aqueous solution following these two different selections is shown in Fig. 4. It is noted that in the logarithmic plots the decay curves show a double sigmoidal shape reporting on the relaxation of the different spin density elements present during the relaxation delay. For example, for the selection of an element proportional to *N*_z_ −16*N*_z_***H***_z_***H***_z_***H***_z_***H***_z_ the faster relaxation of 16*N*_z_***H***_z_***H***_z_***H***_z_***H***_z_ is seen by the first sigmoidal shape and thereafter the relative ratio of the intensity of the four observed lines approaches the 1:2:0:−2:−1 ratio that is characteristic for the in-phase magnetisation. Similarly, for selection of an initial spin density element proportional to 2*N*_z_***H***_z_ + 8*N*_z_***H***_z_***H***_z_***H***_z_ the ratio of the intensity of the four signals approaches 1:1:0:1:1, which is characteristic of the single anti-phase coherence 2*N*_+_***H***_z_.

The decay curves were analysed by evolution of the Liouvillian in the Cartesian basis, **Γ**, during the relaxation delay *T*_relax_. Here the Liouvillian is a function of the two parameters τ_C_ and *R*_1_(***H***_z_) and describes the auto- and cross-correlated relaxation rates within the basis set {*E*/2, ***H***_z_, 2***H***_z_***H***_z_, 4***H***_z_***H***_z_***H***_z_, 8***H***_z_***H***_z_***H***_z_***H***_z_, *N*_z_, 2*N*_z_***H***_z_, 4*N*_z_***H***_z_***H***_z_, 8*N*_z_***H***_z_***H***_z_***H***_z_, 16*N*_z_***H***_z_***H***_z_***H***_z_***H***_z_}. Using a larger basis set that includes also the zero-quantum density elements such as *N*_z_***H***_+_***H***_−_ and 2*N*_z_***H***_+_***H***_−_***H***_z_, did not change the quality of the fit nor did it change the obtained parameters. Briefly, a vector describing the initial state **v**_0_ = {0, 0, 0, 0, 0, *I*_0_(*N*_z_), *I*_0_(2*N*_z_***H***_z_), *I*_0_(4*N*_z_***H***_z_***H***_z_), *I*_0_(8*N*_z_***H***_z_***H***_z_***H***_z_), *I*_0_(16*N*_z_***H***_z_***H***_z_***H***_z_***H***_z_)} was evolved for a time of *T*_relax_. The intensity of the density elements, *E*/2, ***H***_z_, 2***H***_z_***H***_z_, 4***H***_z_***H***_z_***H***_z_, and 8***H***_z_***H***_z_***H***_z_***H***_z_ were assumed to vanish, because phase ϕ_1_ in the pulse sequence of Fig. 1 is phase-cycled {*x*,−*x*} with no concomitant change in the receiver phase, which eliminates contributions from density elements not proportional to *N*_z_ or *N*_y_. After evolving the initial state **v**_0_ for a time of *T*_relax_ the intensities of **v**(*T*_relax_) were converted to intensities of the four lines observed, {*I*_αααα_, *I*_αααβ_, *I*_αβββ_, *I*_ββββ_}. The best-fit model parameters, τ_C_, *R*_1_(***H***_z_), and **v**_0_, were subsequently obtained by a Levenberg-Marquardt least-squared fitting procedure (see Section 5).

Analysis of the decay curves in Fig. 4 gives a correlation time of τ_C_ = 1.63 ps ± 0.03 ps and *R*_1_(***H***_z_) = 4.34 s^−1^ ± 0.02 s^−1^. Moreover, the intensities obtained for the initial states were **v**_0_ = {0, 0, 0, 0, 0, 0.017 ± 0.001, 0.552 ± 0.001, 0.002 ± 0.001, 0.480 ± 0.001, 0.002 ± 0.001} for the data shown in Fig.4A and **v**_0_ = {0, 0, 0, 0, 0, −1.106 ± 0.002, 0.003 ± 0.001, 0.037 ± 0.002, 0.002 ± 0.001, 0.991 ± 0.005} for the data shown in Fig.4B, respectively. It is seen that the obtained selection is very similar to the predicted selection, with *I*_0_(2*N*_z_***H***_z_)/*I*_0_(8*N*_z_***H***_z_***H***_z_***H***_z_) = 1.15 (predicted value = 1) and *I*_0_(*N*_z_)/*I*_0_(16*N*_z_***H***_z_***H***_z_***H***_z_***H***_z_) = −1.12 (predicted value = −1). The small deviation from the predicted values could be a result of relaxation during the selection element and if τ_a_ is slightly different from 1/|4*J*|.

In order to verify the obtained correlation time and proton spin-flip rate a second set of data was obtained at a static magnetic field strength of 16.44 T (700 MHz proton frequency). Here, the same two experiments were recorded as above, that is (i) selecting for an initial state proportional to 2*N*_z_***H***_z_ + 8*N*_z_***H***_z_***H***_z_***H***_z_ and (ii) selecting for an initial state proportional to *N*_z_ – 16*N*_z_***H***_z_***H***_z_***H***_z_***H***_z_. The quality of the data obtained at 16.44 T are similar to those obtained at 11.74 T and simultaneous analysis of the two data set at 16.44 T gives τ_C_ = 1.47 ps ± 0.04 ps and *R*_1_(***H***_z_) = 4.32 s^−1^ ± 0.02 s^−1^. The spin-flip rate obtained at 16.44 T agrees extremely well with the spin-flip rate obtained at 11.74 T. Contributions from the ^15^N chemical shift anisotropy (CSA) relaxation mechanism to the relaxation rates are expected to vanish for the ammonium ion due to its tetrahedral symmetry. The slightly shorter correlation time obtained at 16.44 T compared to 11.74 T confirms that contributions from ^15^N CSA to the relaxation is negligible, since an ^15^N CSA contribution to the longitudinal relaxation rates would lead to larger relaxation rates at higher fields and thus an artificially too long correlation times being obtained at higher fields. Moreover, the correlation time obtained here is in good agreement with the rotational correlation time obtained previously from measurement of in-phase longitudinal *R*_1_(*N*_z_) relaxation rates of ^15^N-ammonium in water and at 276.5 K; τ_C_ = 1.41 ps [5].

*Application to ^15^N-ammonium bound to the 41kDa ATP binding domain of DnaK:* The pulse sequence of Fig. 1 together with the equations for the auto- and cross-correlated relaxation rates within the ^15^NH_4_^+^ spin system provide the basis to characterise the local dynamics and chemical exchange properties of ammonium ions in various environments. The applications above to ^15^N-ammonium in an acidic aqueous solution provide the correlation time of the ammonium ion and thus provide a validation of the pulse sequence shown in Fig. 1 for the measurement of longitudinal relaxation rates of ^15^NH_4_^+^ longitudinal spin density elements. The correlation time for ammonium ions in various solvents have been characterised [6], however the correlation time of ammonium ions within specific monovalent cation binding-sites in proteins have not been characterised previously. Previous applications [19], [20] have shown that ^15^N-ammonium within potassium binding-sites in medium-large proteins can be probed using ^15^N-^1^H correlation spectra and the pulse sequence presented above in Fig. 1 therefore opens up for the possibility of measuring longitudinal relaxation rates of ^15^N-ammonium within potassium-binding sites in medium-large proteins.

The activity of the bacterial Hsp70 homologue DnaK relies on the binding of two potassium ions, where the two potassium ions in the ATP binding domain have been shown to be crucial for the ATP cycle [22]. Of interest here is that potassium can be substituted by ammonium with the enzyme retaining more than half of its activity [22]. Previous applications have shown that ^15^N-ammonium within the two potassium binding sites of a 41 kDa domain of DnaK can be probed using ^15^N-^1^H correlation spectra, when ADP and inorganic phosphate are added to create an environment that protects the ammonium ion from the bulk solvent. An initial application below to ^15^N-ammonium bound to one of the two potassium binding-sites in DnaK will illustrate the applicability of the method of measuring longitudinal relaxation rates of ^15^N-ammonium in medium-large proteins.

**Fig. 5:**
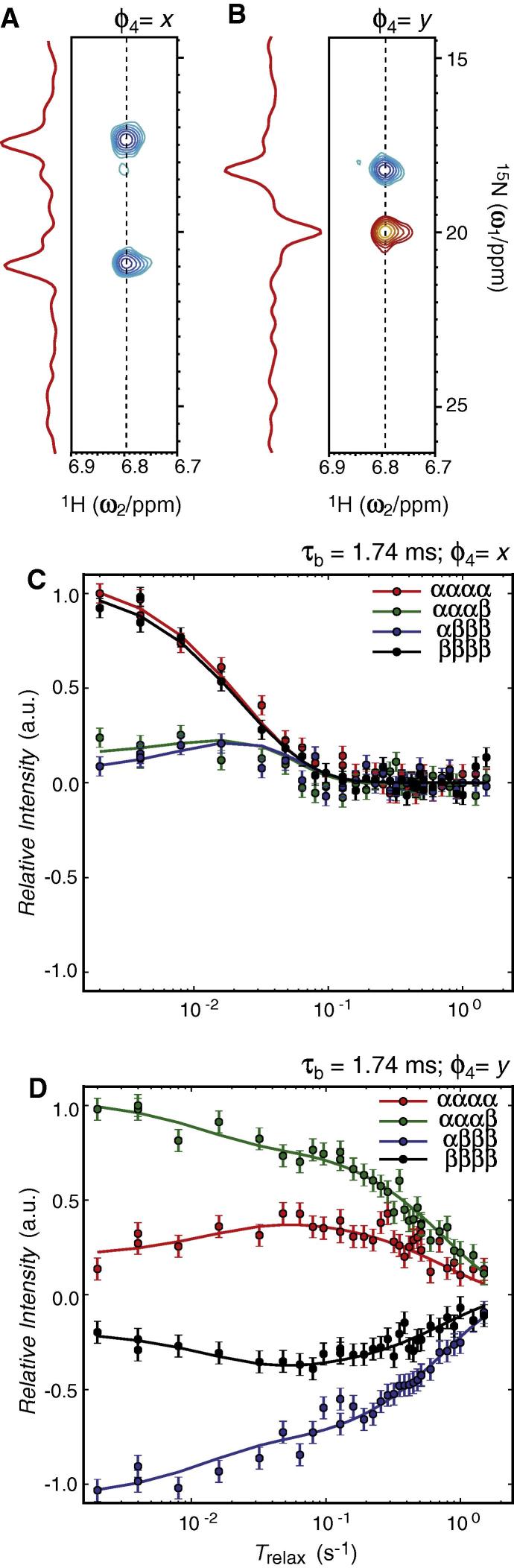
Characterising ^15^N longitudinal relaxation of ^15^NH_4_^+^ bond to a ∼41 kDa domain of DnaK at a field of 18.79 T and a temperature of 278 K. (A) ^15^N-^1^H correlation spectrum obtained after selecting for 2*N*_+_***H***_z_ + 8*N*_+_***H***_z_***H***_z_***H***_z_. (B) ^15^N-^1^H correlation spectrum obtained after selecting for *N*_+_ − 16*N*_+_***H***_z_***H***_z_***H***_z_***H***_z_ using the pulse sequence of Fig 1. (C) Relaxation decay curve obtained following the selection in A. (D) Relaxation decay curve obtained following the selection in B. Shown in solid lines are the global fit of all the data shown in C and D.

Selection of the initial state and measurements of longitudinal relaxation rates of ^15^N-ammonium in DnaK at 18.79 T (800 MHz proton frequency) and at 278 K is shown in Fig. 5.

As for the application to ^15^N-ammonium in acidic aqueous solutions shown above two relaxation experiments were carried out, that is, selecting for an initial state proportional to 2*N*_z_***H***_z_ + 8*N*_z_***H***_z_***H***_z_***H***_z_ (Fig.5A) and selecting for an initial state proportional to *N*_z_ – 16*N*_z_***H***_z_***H***_z_***H***_z_***H***_z_ (Fig.5B). A simultaneous analysis of the two set of data and neglecting chemical exchange provides a proton spin-flip rate of *R*_1_(***H***_z_) = 19.7 s^−1^ ± 1.4 s^−1^ and an effective correlation time of τ_C,eff_ = 66.3 ± 3.5 ps. These model parameters correspond to auto-relaxation rates of *R*_1_(*N*_z_) = 1.30 ± 0.07 s^−1^, *R*_1_(2*N*_z_***H***_z_) = 26.6 ± 1.5 s^−1^, *R*_1_(4*N*_z_***H***_z_***H***_z_) = 48.6 ± 2.9 s^−1^, *R*_1_(8*N*_z_***H***_z_***H***_z_***H***_z_) = 68.0 ± 4.3 s^−1^, and *R*_1_(16*N*_z_***H***_z_***H***_z_***H***_z_***H***_z_) = 85.5 ± 5.7 s^−1^. Moreover, the intensities obtained for the initial states are **v**_0_ = {0, 0, 0, 0, 0, 0.028 ± 0.027, 0.632 ± 0.017, −0.018 ± 0.024, 0.432 ± 0.021, 0.011 ± 0.077} for the data shown in Fig.5C and **v**_0_ = {0, 0, 0, 0, 0, −1.693 ± 0.040, 0.021 ± 0.016, 0.091 ± 0.026, −0.002 ± 0.020, 0.276 ± 0.086} for the data shown in Fig.5D, respectively. The large proton spin-flip rate causes relaxation during the selection element, such that the selections shown in Fig.5A and B are not exactly proportional to 2*N*_z_***H***_z_ + 8*N*_z_***H***_z_***H***_z_***H***_z_ and *N*_z_ – 16*N*_z_***H***_z_***H***_z_***H***_z_***H***_z_, respectively. This becomes particularly apparent for the selection of *N*_z_ – 16*N*_z_***H***_z_***H***_z_***H***_z_***H***_z_ (Fig.5B and D), where the fast relaxation of the quadruple anti-phase element leads to a ratio of *I*_0_(16*N*_z_***H***_z_***H***_z_***H***_z_***H***_z_)/*I*_0_(*N*_z_)∼−0.16 instead of −1. It should be stressed that a determination of the initial state is included in the least-squared analysis via the model parameter **v**_0_ and as such the different selections serve merely as a means of providing initial states that are different enough to allow for an accurate determination of both *R*_1_(***H***_z_) and τ_C,eff_.

It is interesting to note that the effective rotational correlation time obtained here for the ammonium ion is very similar to the rotational correlation time of a methyl group within a protein environment, which has been found to be between 25 ps ≲ τ_Me_ ≲ 125 ps, depending on residue type and temperature [29], [30], [31]. A previous investigation of the dynamics of lysine side chains has shown that the correlation time for the rotation about the threefold axis of the lysine —NH_3_^+^ group reports on hydrogen bonding [32]. The potassium binding sites in DnaK are lined with negative charges from aspartic acid side chains and phosphate groups and hydrogen-bonding and/or salt-bridging is therefore possible and could explain the ca. 40 times longer correlation time for DnaK-bound ammonium compared to free ammonium.

It was assumed above that the rotational correlation function for ^15^NH_4_^+^ bound to DnaK can be described accurately with one effective correlation time and chemical exchange events, for example dissociation of ^15^NH_4_^+^ from the binding site, were neglected. The dependence of the ^15^NH_4_^+^ longitudinal relaxation rates on temperature and the dependence of the rates on the static magnetic field strength are still to be explored. Such future explorations will, for example, give information about whether the correlation function for the rotational diffusion of ammonium ions bound to proteins is accurately described by one correlation time, τ_C,eff_, or if more elaborate models for the correlation function need to be employed. The temperature dependence of the derived relaxation rates will aid to elucidate possible chemical exchange events such as dissociation of the ammonium ion.

## 4 Conclusions

In summary, NMR pulse schemes have been developed to both select different longitudinal spin density matrix elements of ^15^N-ammonium and also to obtain the longitudinal ^15^N relaxation rates of these longitudinal spin density elements. An initial application to ^15^NH_4_^+^ in an acidic aqueous solution was used to validate the pulse scheme and the new method to derive the effective correlation time of the ^15^NH_4_^+^ spin system.

An application of the derived pulse scheme to probe the dynamics of enzyme-bound ammonium ions was subsequently described, where in particular the possibility of characterising ^15^N-ammonium bound to a 41 kDa domain of DnaK at 278 K shows the very general applicability of the method. For such a system, the protein itself is expected to have a rotational correlation time of approximately 36 ns at 278 K, thus confirming that the derived method is applicable to characterise potassium binding-site in medium-large proteins.

The pulse scheme and method presented here provides an avenue for further investigations of protein-bound ammonium ions to elucidate the properties of potassium-binding sites in large proteins and also characterise the kinetic aspects of monovalent cation binding in such systems.

## 5 Material and methods

*Sample preparations:* A sample of ^15^NH_4_^+^ in an acidic aqueous solution was prepared by dissolving 21 mg of ^15^NH_4_Cl in 2 ml of 100% H_2_O containing 50 mM sodium phosphate, 2 mM EDTA, 25 mM NaCl, 2 mM NaN_3_. The pH was subsequently adjusted to 2.82 to slow the chemical exchange of the ammonium protons with the H_2_O. The NMR sample of the ATP binding domain of DnaK from *Thermus thermophilus* was prepared as explained previously[19]. The protein concentration was ∼100 μM in 100% H_2_O containing 150 mM ^15^NH_4_Cl, 0.5 mM ADP, 50 mM (NH_4_)H_2_PO_4_, 5 mM MgCl_2_, 1 mM DTT, 1 mM NaN_3_ and 75 mM Tris, pH 7.5.

*NMR experiments:* The NMR experiments were performed on a Bruker Avance III 500 MHz (11.7 T) spectrometer using an HCN Prodigy probe, a Bruker Avance III 700 MHz (16.4 T) spectrometer using a TCI cryogenic inverse triple-resonance probe, and a Bruker Avance III HD 800 MHz (18.8 T) spectrometer equipped with a cryogenic inverse triple-resonance TCI probe. All spectra were measured with an external D_2_O reference insert (Wilmad coaxial insert Z278513 (Sigma–Aldrich)), such that no D_2_O was added to the sample buffer.

The longitudinal relaxation rates of free ^15^N-ammonium at pH 2.82 were measured at a static magnetic field of 11.7 T and 16.5 T using the pulse scheme shown in Fig. 1. For the spectra showing the initial states in Fig. 3, an inter-scan delay of 1 s was used, *T*_relax_ = 4 ms, and 32 complex point were acquired in the indirect ^15^N frequency dimension. Relaxation delays *T*_relax_ of {0.004 s, 0.010 s, 0.020 s, 0.020 s, 0.030 s, 0.040 s, 0.050 s, 0.060 s, 0.080 s, 0.100 s, 0.120 s, 0.140 s, 0.160 s, 0.200 s, 0.300 s, 0.400 s, 0.500 s, 0.600 s, 0.800 s, 0.800 s, 1.000 s, 2.000 s, 5.000 s, 10.00 s, 15.00 s} were used for all relaxation experiments. Eight scans were acquired for each FID leading to a net acquisition time of 10 h for each initial state {τ_b_,ϕ_4_}.

Longitudinal relaxation rates of ^15^N-ammonium bound to DnaK were measured at a static magnetic field strength 18.8 T. A total of 32 complex points were acquired in the indirect ^15^N frequency dimension and 32 *T*_relax_ relaxation delays were used: {0.002 s, 0.004 s, 0.004 s, 0.008 s, 0.016 s, 0.032 s, 0.048 s, 0.064 s, 0.080 s, 0.096 s, 0.128 s, 0.128 s, 0.160 s, 0.192 s, 0.224 s, 0.256 s, 0.288 s, 0.320 s, 0.352 s, 0.384 s, 0.416 s, 0.448 s, 0.480 s, 0.512 s, 0.512 s, 0.600 s, 0.700 s, 0.800 s, 0.900 s, 1.000 s, 1.250 s, 1.500 s} for each initial state {τ_b_,ϕ_4_}. An inter-scan delay of 1 s was used and 40 scans were obtained for each FID, leading to total acquisition time of 34 h for each initial state.

*Data analysis:* All spectra were processed using nmrPipe [33] and signal intensities were quantified using the program FuDA [34] by assuming a common line shape for a given cross-peak during a relaxation series as described previously [15].

Relaxation decay curves for the four lines, *I*_αααα_(*T*_relax_), *I*_αααβ_(*T*_relax_), *I*_αβββ_(*T*_relax_), and *I*_ββββ_(*T*_relax_) as a function of *T*_relax_ were analysed using the propagation of the full Liouvillian and the best-fit model parameters were obtained by minimisation of the target function:

(6) $$\chi^{2}(\tau_{C}\text{,}R_{1}(H_{z})\text{,}v_{0})=\underset{\lambda =\{\alpha \alpha \alpha \alpha \text{,}\ldots \text{,}\beta \beta \beta \beta \}}{\sum }\underset{T_{\mathrm{relax}}}{\sum }{\left(I_{\lambda }^{\mathrm{calc}}(T_{\mathrm{relax}})-I_{\lambda }^{\exp}(T_{\mathrm{relax}})\right)}^{2}/\sigma_{\exp}^{2}$$

where **v**_0_ is the initial state described in the main text. The first sum is over the four lines, αααα, αααβ, αβββ, and ββββ, and the second sum is over the different relaxation delays *T*_relax_. Moreover, $I_{\lambda }^{\exp}(T_{\mathrm{relax}})$ and $\sigma_{\exp}$ are experimental intensities of the four lines observed in ^15^N-^1^H correlation spectra and their uncertainties, respectively, $I_{\lambda }^{\mathrm{calc}}(T_{\mathrm{relax}})$ are calculated intensities obtained by numerical propagation of the initial state using the Liouvillian, **Γ**.

Calculated intensities, $I_{\lambda }^{\mathrm{calc}}(T_{\mathrm{relax}})$ were obtained by first calculating a vector **v**(*T*_relax_), which describes the time-dependence of the population of the longitudinal coherences, {*I*(*N*_z_), … *I*(16*N*_z_***H***_z_***H***_z_***H***_z_***H***_z_)}:

(7) $$v(T_{\mathrm{relax}})=\exp(-\Gamma (\tau_{\text{C}}\text{,}R_{1}(H_{\text{z}}))T_{\mathrm{relax}})v_{0}$$

Subsequently, the intensities of the four lines observed in the ^15^N-^1^H correlation spectra were calculated as described previously [20]:

(8) $$\left(\begin{array}{c} I_{\alpha \alpha \alpha \alpha }\\ I_{\alpha \alpha \alpha \beta }\\ I_{\alpha \beta \beta \beta }\\ I_{\beta \beta \beta \beta } \end{array}\right)=\left(\begin{array}{ccccc} -1/4 & 1 & -3/2 & 1 & -1/4\\ -1/2 & 1 & 0 & -1 & 1/2\\ 1/2 & 1 & 0 & -1 & -1/2\\ 1/4 & 1 & 3/2 & 1 & 1/4 \end{array}\right)\left(\begin{array}{c} I(N_{z})\\ I(2N_{z}H_{\text{z}})\\ I(4N_{z}H_{\text{z}}H_{\text{z}})\\ I(8N_{z}H_{\text{z}}H_{\text{z}}H_{\text{z}})\\ I(16N_{z}H_{\text{z}}H_{\text{z}}H_{\text{z}}H_{\text{z}}) \end{array}\right)$$

Finally best-fit model parameters were determined by minimising the target function χ^2^ in Eq. (6) using in-house written software based on the LMFIT python library [35].
